# The segmentation of nanoparticles with a novel approach of HRU^2^-Net^†^

**DOI:** 10.1038/s41598-025-86085-w

**Published:** 2025-01-16

**Authors:** Yu Zhang, Heng Zhang, Fengfeng Liang, Guangjie Liu, Jinlong Zhu

**Affiliations:** School of Computer Science and Technology, Changchun Normal University, Changchun, 130032 China

**Keywords:** Nanoparticles, Deep learning, HRU^2^-Net^†^ model, Image segmentation, Nanoscience and technology, Nanoscale materials

## Abstract

Nanoparticles have great potential for the application in new energy and aerospace fields. The distribution of nanoparticle sizes is a critical determinant of material properties and serves as a significant parameter in defining the characteristics of zero-dimensional nanomaterials. In this study, we proposed HRU^2^-Net^†^, an enhancement of the U^2^-Net^†^ model, featuring multi-level semantic information fusion. This approach exhibits strong competitiveness and refined segmentation capabilities for nanoparticle segmentation. It achieves a Mean intersection over union (MIoU) of 87.31%, with an accuracy rate exceeding 97.31%, leading to a significant improvement in segmentation effectiveness and precision. The results show that the deep learning-based method significantly enhances the efficacy of nanomaterial research, which holds substantial significance for the advancement of nanomaterial science.

## Introduction

Nanoparticles, with their extremely small size and unique electrical, optical, magnetic and thermal properties^1^, have great potential for applications in agricultural production, biomedicine, new energy sources and aerospace^2^, as well as providing the material science basis for many advanced technologies. Given the intricate nature and nanoscale dimensions of nanoparticles, investigating them demands the deployment of highly accurate instruments, such as optical microscopes, atomic force microscopes and electron microscopes^3^. Extracting nanoparticle data from large-scale equipment and distilling valuable information is labor-intensive and inefficient. Moreover, the complex information inherent in nanoparticles is challenging to extract using manual methods, leading to a slow pace in the research and application of nanoparticles^4^.

In recent years, deep learning technology has been successfully applied to many scientific fields^5–12^. At the same time, deep learning technology has been well applied in fields such as new material prediction, material structure and scale determination^13^. Recently, many methods have emerged for the segmentation problem of nanoparticles. Zhijian Sun et al. proposed a general framework based on deep learning and used a lightweight deep learning network (NSNet) to achieve segmentation, shape extraction, and statistical analysis of nanoparticles, with an accuracy rate of 86.2% and is capable of processing 11 SEM/TEM images per second on an embedded processor^14^. Khuram Faraz t al. used deep learning and multi-target tracking to implement an procedure to automatically track, scan and observe nanoparticlese, with MOTA reaching 99.5%, MOTP reaching 94.9%, FP being 0, and FN being 9, indicating high accuracy and precision.^15^. Zelin Wang et al. designed a Transformer Enhanced Segmentation Network (TESN) using a hybrid CNN Transformers architecture based on Transformer^13^ and Mask R-CNN^16^ to accurately segment and measure nanoparticles, with an error range of TESN is from 0.38 to 3.52%^17^. Leonid Mill et al. solved the problem of nanoparticle data volume and labeling by synthesizing images, which can train state-of-the-art deep neural networks.For SiO2 nanoparticles, the F1 score of U-Netreal is 0.950, while the F1 score of U-Netsim is 0.930. For TiO2 nanoparticles, the F1 score of U-Netreal is 0.943, and the F1 score of U-Netsim is 0.923^18^. Current methods for segmenting nanoparticles primarily rely on data statistics, machine learning, and a limited number of deep learning techniques. Given the significant variability in nanoparticle shapes, with many tending to aggregate, the application of more traditional network models like U-Net^19^., SegNet^20^., and ResNet^21^. For nanoparticle segmentation lacks universality and cutting-edge advancements, which is detrimental to the accurate segmentation of these particles.

In this work, we propose a HRU^2^-Net^†^ model based on the U^2^-Net^†^^22^ and U-HRNet^23^ models, which has a new U-shapled of multi-level semantic information fusion module and can be used to analyse titanium dioxide nanoparticle image data obtained from scanning electron microscopy.Our main contributions are summarized as follows:

- A new U-shaped of multi-level semantic information fusion module is proposed, which is able to give a precision segmentation results.
- The novel deep learning model is performed for nanoparticles segmentation, which provides a new solution in nanomaterial research.
- Results from the experiments on the nanoparticles dataset show that the model integrates feature maps of different resolutions, capturing global context information while preserving high-resolution details, making the model’s segmentation of details more refined.

## Methods

### ***HRU***^***2***^***-Net***^***†***^*** architecture***

**Fig. 1:**
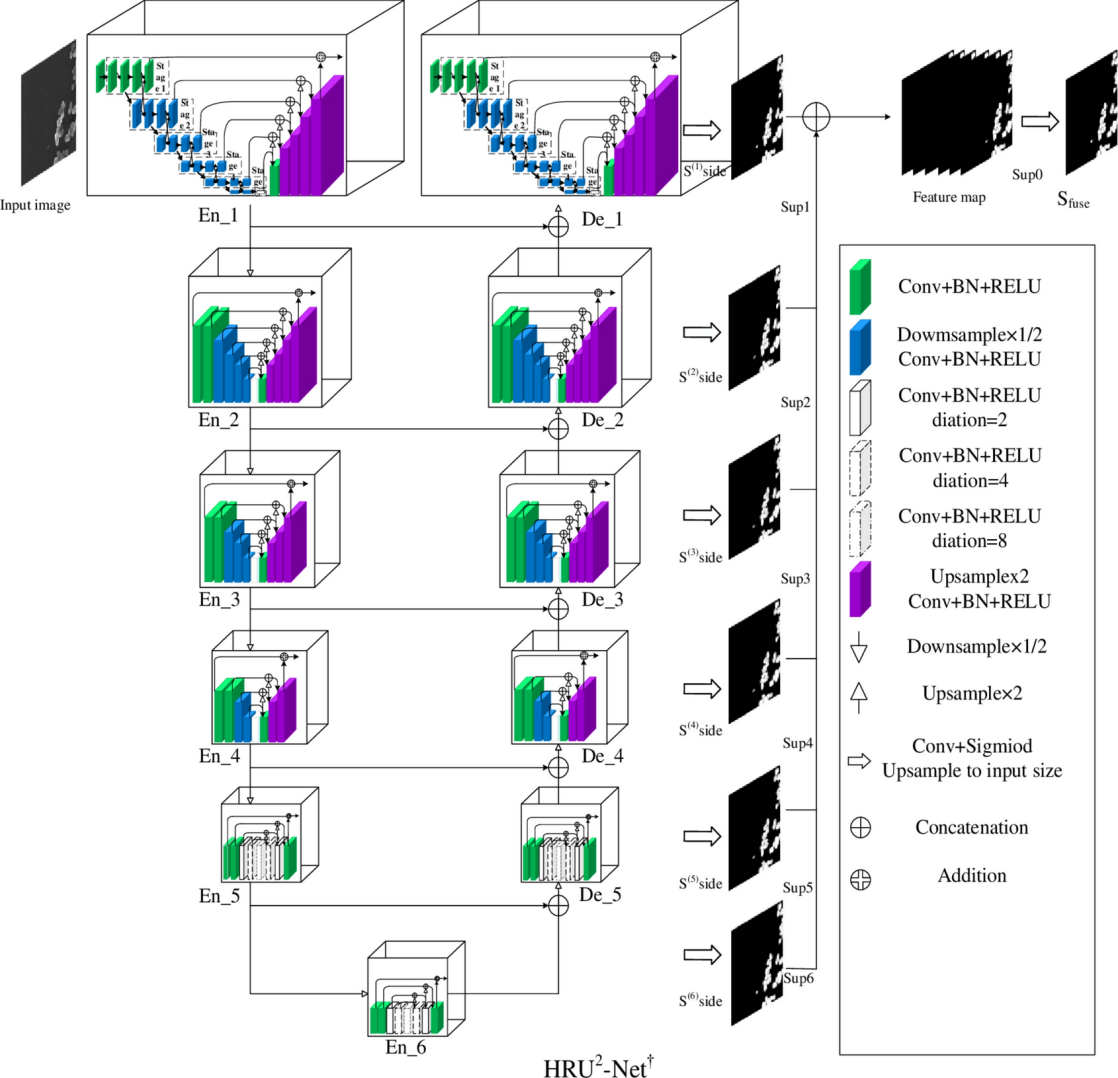
HRU^2^-Net^†^ model structure.

The HRU^2^-Net^**†**^ model combines the idea of multi-level semantic information fusion design and the resolution improvement of feature map on the basis of U^2^-Net^**†**^ model. A multilevel information fusion module is added to the top-level encoder and decoder of the nested U-shaped structure, which improves the resolution and segmentation speed of the feature map. The overall structure is shown in Fig. 1. The En_1 and De_1 are the designed multi-level semantic information fusion modules (MSIF). The MSIF module is consistent with U^2^-Net^**†**^ model in the whole layer, and it can be applied to the En_2, En_3, De_2, De_3 parts as needed. The MSIF module can connect the semantic information between the feature maps of different resolutions, which makes the semantic information flow between different resolutions better. Therefore, the segmentation ability of the model image is enhanced, the model computation is reduced, and the resolution of the feature image is improved.

**Fig. 2:**
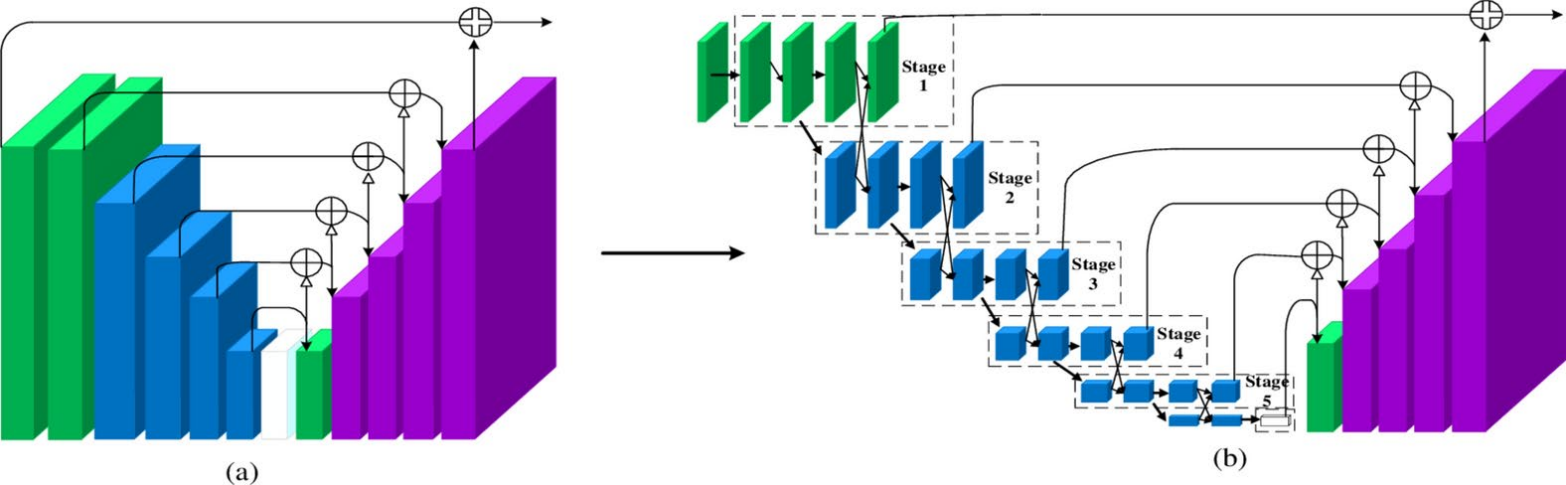
The En_1 and De_1 structures (**a**) The En_1, De_1 structure of the U^2^-Net† model (**b**) Our U-shaped of multi-level semantic information fusion module (UMSIF).

The MSIF module can bridge the semantic information between feature maps of varying resolutions, facilitating better circulation of semantic information across different scales. This enhances the model’s image segmentation capabilities, reduces the computational load, and improves the resolution of the feature maps. Additionally, the MSIF module is incorporated only in the encoder part of the high-resolution structural design, which results in a more compact model size and consequently improved operational efficiency. The structure of the model is illustrated in Fig. 2.

Figure 2a displays the original En_1, De_1 structure of the U2-Net† model. Figure 2b is the U-shaped MSIF module designed in this paper, which is applied to the En_1, De_1 part of the HRU2-Net† model. The U-shaped MSIF module is consistent with the U2-Net† model in terms of the overall number of layers, and its structure is designed for the fusion of upper and lower level semantic information. Moreover, this enhancement can be applied to the En_2, En_3, De_2, De_3 sections as required.

### Loss function

Depending on the size and category of the dataset this paper uses a cross entropy loss function^24^ to represent the degree of difference between the predicted and actual data, which is expressed as.

1 $$Loss = - \frac{1}{N}\sum\limits_{i} {\sum\limits_{c = 0}^{M} {\mathop y\nolimits_{ic} } } \log (\mathop p\nolimits_{ic} )$$

In the formula, Pic is the predicted probability that the observed sample i belongs to category c, Yic is the sign function, if the true category of sample i is equal to c take 1 otherwise take 0. M is the number of label types, N is the total number of pixel points.

### Training setup

**Fig. 3:**
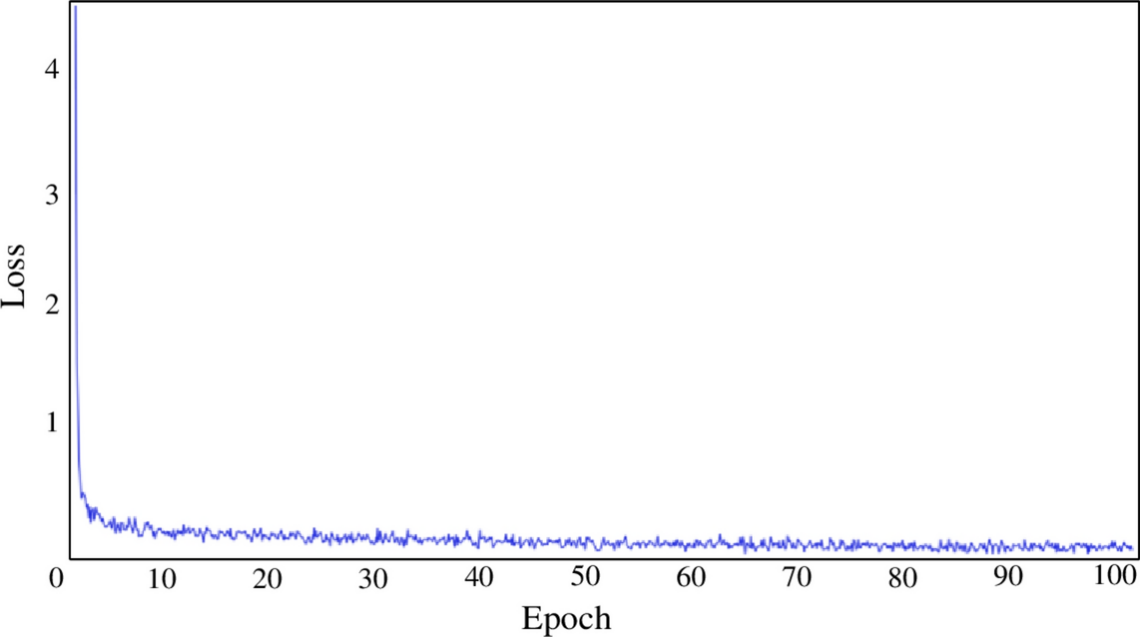
Loss function curve.

During the training process, the nanomaterial images were resized to 512 × 512 pixels. ResNet, HRNet^25^ and STDC^26^ were used as the backbone networks of the partial semantic segmentation model. When pre-training the network model, we observed that the loss function converged around 50 iterations, hence we set the number of training iterations to 100, as depicted in Fig. 3. We used the cross-entropy loss function to represent the degree of difference between the predicted and actual data, set the initial learning rate to 0.01 and used the learning rate decay method for gradual decay. The optimization method is based on SGD^26^, with a batch size of 16 and random initialization, and the whole experiment takes about 400 h. Table1 illustrates the HRU 2-Net network structure configuration. The experimental equipment is based on 64-bit Windows 11 operating system and PaddleSeg^27^ is used to build the training and testing network. The detailed configurations are as follows: Anaconda3, PaddlePaddle2.4.0, Paddleseg2.7.0, OpenCV4.6.0, Cuda11.2 and Cudnn8.2.

**Table 1:** HRU2-Net† Network Structure Configuration.

Level	Module	Operate	Output size
	Input	Input image	512 × 512
Encoder			
Layer 1	En_1	MSIF	256 × 256
Layer 2	En_2	Down-sampling × 1/2, Conv + BN + ReLU	128 × 128
Layer 3	En_3	Down-sampling × 1/2, Conv + BN + ReLU	64 × 64
Layer 4	En_4	Down-sampling × 1/2, Conv + BN + ReLU	32 × 32
Layer 5	En_5	Down-sampling × 1/2, Conv + BN + ReLU	16 × 16
Layer 6	En_6	Down-sampling × 1/2, Conv + BN + ReLU	8 × 8
Decoder			
Layer 5	De_1	MSIF	16 × 16
Layer 4	De_2	Up-sample × 2, Conv + BN + ReLU	32 × 32
Layer 3	De_3	Up-sample × 2, Conv + BN + ReLU	64 × 64
Layer 2	De_4	Up-sample × 2, Conv + BN + ReLU	128 × 128
Layer 1	De_5	Up-sample × 2, Conv + BN + ReLU	256 × 256
	Output	Feature map	512 × 512

## Results

### Datasets

**Fig. 4:**
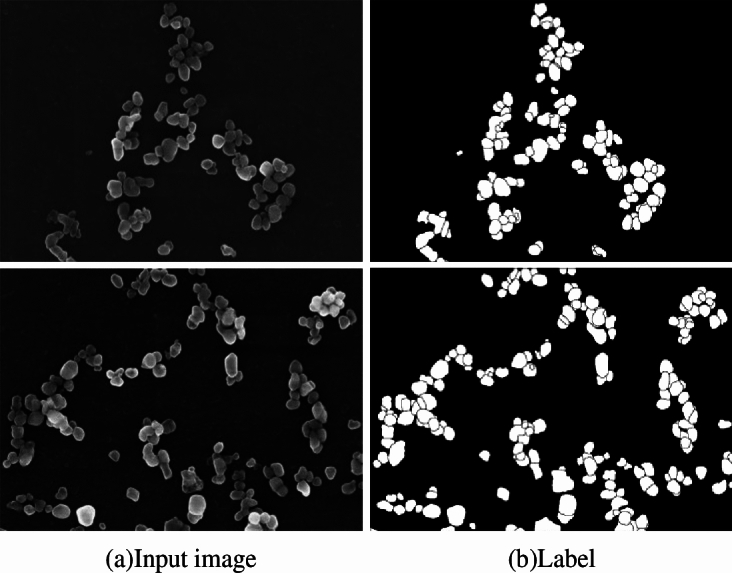
Image of titanium dioxide nanoparticles (**a**) original data image (**b**) The annotated image of (**a**).

The nanoparticle dataset was obtained from the open source project of Bastian Rühle et al^28^, whose image data were generated and annotated with the aid of a GAN network and could be used for the training of a convolutional neural network. The titanium dioxide nanoparticles were labeled as white and the background as black, as shown in Fig. 4. The image data was cropped to a total of 200 images of 512 × 512 pixels and expanded using the Data Expansion and Enhancement tool^29^ as required. Finally, the processed image data were randomly divided into a training data set and a test data set in the ratio of 8:2, with 2000 training data sets and 500 test data sets.

### ***The performance of the HRU***^***2***^***-Net***^***†***^*** on nanoparticles***

Nanoparticles, due to their minuscule size, diverse and intricate structures, pose numerous challenges in research and preparation. Meanwhile, the physical and chemical properties of various nanoparticles are quite different from each other, and some nanoparticles even have toxic side effects. Therefore, it is crucial to select a reliable semantic segmentation model to accomplish the image segmentation task of nanoparticles. A network model for practical application should not only consider its accuracy, but also its robustness, scalability and resource dependence. To comprehensively evaluate these network models, seven evaluation metrics were used in this paper^30–32^: (1) Mean intersection over union (MIoU), (2) Accuracy, (3) Kappa coefficient (Kappa), (4) Dice coefficient (Dice), (5) Intersection over union (IoU).

(1) MIoU^30^ is a semantic segmentation metric that calculates the average of the ratio of intersection and concatenation of all classes. Its public representation is as follows.

2 $$MIoU = \frac{1}{{N_{cls} }}\sum\nolimits_{x = 1}^{{N_{cls} }} {\frac{{N_{xx} }}{{\sum\nolimits_{y = 1}^{{N_{cls} }} {N_{xy} } + \sum\nolimits_{y = 1}^{{N_{cls} }} {N_{yx} } - N_{xx} }}}$$

According to the confusion matrix, where Ncls denotes the total number of categories, Nxx denotes true positives, Nxy denotes false positives, Nyx denotes false negatives, and Nyy denotes true negatives.

(2) Accuracy^30^ is a metric used to evaluate classification models, i.e. the proportion of the total number of correct model predictions, with the following formula.

3 $$Accuracy = \frac{TP + TN}{{TP + TN + FP + FN}}$$

According to the confusion matrix, where TP is true positive, TN is true negative, FP is false positive and FN is false negative.

(3) Kappa coefficient^32^ is an indicator of consistency test, which refers to whether the model prediction results and the actual classification results are consistent, and it can be used to measure the classification effect. Its formula is as follows.

4 $$Kappa = \frac{{\mathop p\nolimits_{o} { - }\mathop p\nolimits_{e} }}{{1 - \mathop p\nolimits_{e} }}$$

where Po is the sum of the number of correctly classified samples in each category divided by the total number of samples, i.e. the overall classification accuracy. Pe is the "sum of the products of the actual and predicted numbers" for each of the categories, divided by the "square of the total number of samples".

(4) Dice^31^ is a set similarity measure function, which is used to calculate the similarity of two samples and is often used to evaluate the goodness of segmentation algorithms. Its public expression is as follows.

5 $$Dice = \frac{2|A \cap B|}{{|A| + |B|}}$$

where |AB| is the intersection between A and B, and the subtables |A| and |B| denote the number of elements of the sum. The factor of 2 in the numerator is due to double counting of the common elements between A and B in the denominator.

(5) IoU^31^ represents the result of dividing the overlapping part of two regions by the aggregated part of the two regions, which is one of the semantic segmentation metrics. The formula is expressed as follows.

6 $$IoU = J(A,B) = \frac{|A \cap B|}{{|A \cup B|}}$$

It is defined as the area of the intersection between the predicted segmented image A and the truth image B, divided by the area of the union between the two images, with a value ranging from 0 to 1.

**Fig. 5:**
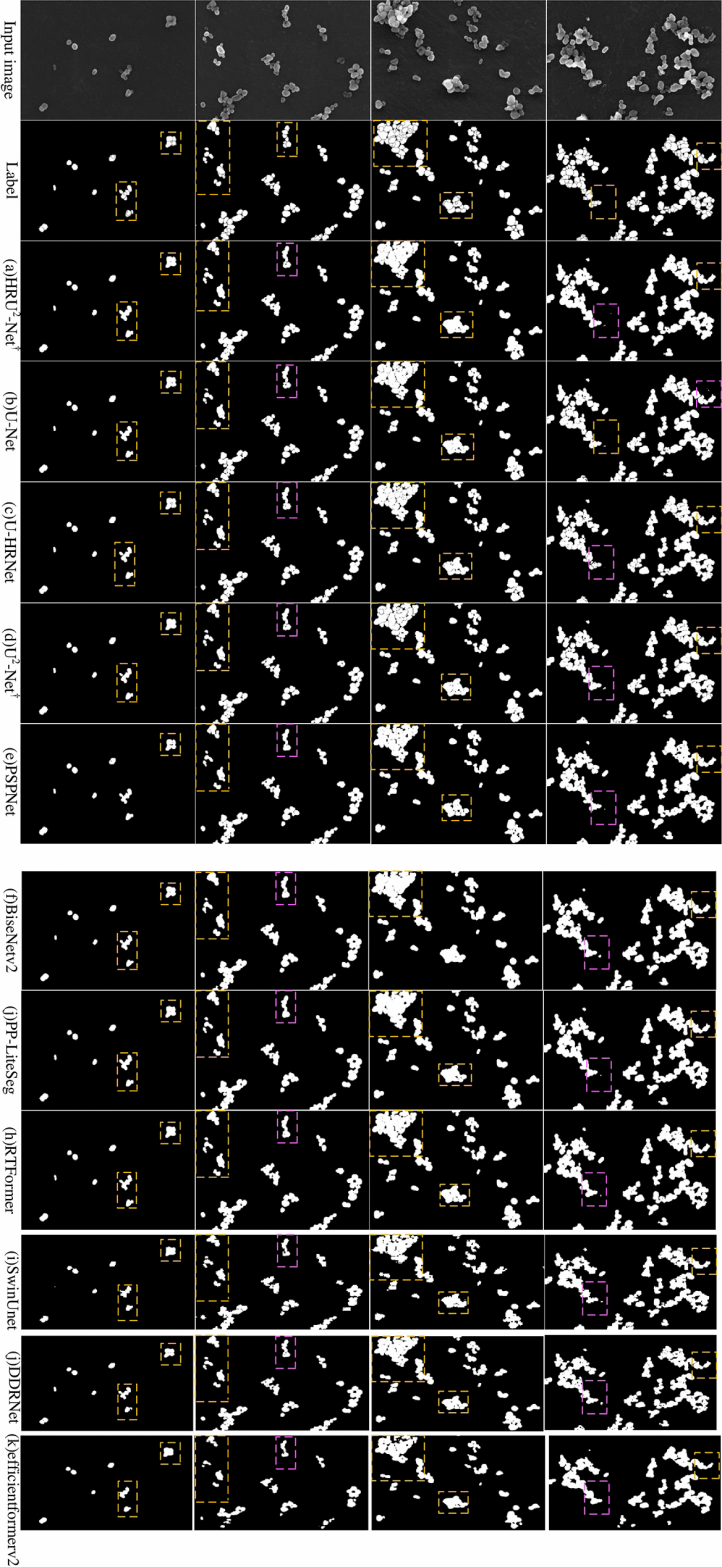
Nanoparticle segmentation results.

UMSIF is a cutting-edge deep learning technology that captures image features across multiple spatial scales, endowing the model with the ability to understand both local details and global context of an image. The core advantage of this technology lies in its capability to traverse point cloud features across different geometric radii, capturing rich semantic information from a local perspective, reducing reliance on single semantic segmentation results, and thereby lowering the risk of bias. UMSIF integrates and aggregates multi-scale semantic information through graph structures, effectively handling complex spatial relationships, and extracting features from both global and local contexts. The combination of global and local features provides the model with a more comprehensive image representation, significantly enhancing its accuracy and robustness in object detection and segmentation tasks. Through the detailed data and visual presentation in Table 1 and Fig. 5, we can clearly observe the effects of UMSIF in capturing and fusing multi-level semantic information, and these results further confirm the significant effectiveness of UMSIF in enhancing model performance.

Table 2 lists the evaluation data of the deep learning based semantic segmentation methods on the nanoparticle dataset. The results show that the MIoU of all models is above 83%, the accuracy is around 97%, the Kappa coefficient is above 83%, and the dice coefficient is above 91%, which indicates that deep learning-based semantic segmentation models exhibit superior segmentation performance and enhanced accuracy.The MIoU of the classical segmentation models, such as U-Net and PSPNet^33^, is around 86%. This is due to the fact that they employ deeper convolutional neural networks, which to some extent enhance the accuracy. However, the larger model size is not conducive for deployment, and there are also efficiency-related drawbacks. U^2^-Net^†^, DDRNet^34^, BiseNetV2^35^, PPLiteSeg, Efficientformerv2^36^ and RTFormer^37^ are lightweight network models, which are small for deployment and transformed segmentation environments, with MIoUs in the range of 83%-87%. HRU-Net uses HRNet as the backbone network, and its model is large in size and runs slowly with an MIoU of 87.21%. We design an improved HRU2-Net† model characterized by its compact size and rapid segmentation capabilities. With a MIoU of 87.37%, it outperforms other models, demonstrating superior overall performance and strong competitiveness.

**Table 2:** Prediction results for nanoparticle data on each model.

Models	Backbone	GFOPs	Params	MIoU (%)	Acc (%)	Kappa (%)	Dice (%)
HRU^2^-Net^†^	–	**42.60**	**6.57**	**87.37**	**97.31**	**85.95**	**92.98**
U-HRNet	HRNet48	–	–	87.21	97.49	85.72	95.86
U^2^-Net^†^	–	48.77	4.36	87.29	97.47	85.83	92.91
U-Net	–	124.31	51.14	86.65	97.08	85.07	92.53
PSPNet	ResNet50	265.5	259.03	86.72	97.33	85.11	92.56
DDRNet	DdrNet_23	17.93	77.00	85.28	97.01	83.28	91.64
PPLiteSeg	STDC2	9.04	46.07	85.77	97.12	83.91	91.95
RTFormer	–	16.90	64.36	85.77	97.01	83.92	91.96
Bisenetv2	–	8.06	8.88	83.96	96.48	81.61	90.80
SwinUnet	–	–	–	84.07	96.40	96.40	92.22
Efficientformerv2	–	–	–	84.57	96.61	82.42	91.20

Significant values are in bold.

As shown in Fig. 5, the actual segmentation effect images for each model are displayed, where the yellow box indicates a comparison of segmentation details, and the pink box highlights the presence of tiny regions that have not been segmented. It can be observed from the figure that the HRU^2^-Net^†^ model is capable of accurately segmenting the tiny nanoparticles within the image during nanoparticle segmentation, and at the same time, the model exhibits no segmentation errors The HRU2-Net† model is capable of achieving fine segmentation for nanoparticles of varying morphologies. Among them, the U2-Net† model excels over the DDRNet, BiseNetV2, PP-LiteSeg^38^, and RTFormer models in terms of segmentation details. Regarding boundary clarity, the DDRNet and PP-LiteSeg models exhibit poor segmentation, while the BiseNetV2, RTFormer, SwinUnet, and EfficientFormerV2 models suffer from a significant lack of segmentation details.The U2-Net† model exhibits some areas that are not fully segmented, whereas the DDRNet and PP-LiteSeg models display a more significant number of unsegmented regions. Moreover, the BiseNetV2 and RTFormer models have a major parts failing to be segmented. In terms of segmentation failures, the U2-Net† model experiences minor segmentation issues in the image boundary regions, while the DDRNet, BiseNetV2 and PP-LiteSeg models demonstrate fewer segmentation failures. These findings indicate that our improved HRU2-Net†model exhibits superior adaptability and segmentation capabilities for nanoparticles, which is of greater significance for broadening the morphological analysis of nanoparticles and achieving a more efficient workflow.

## Conclusions

In the nanoparticle segmentation experiments, we used a variety of state-of-the-art semantic segmentation models as well as our improved HRU2-Net† model based on nanoparticle characteristics. In order to validate the deep learning-based semantic segmentation technique on nanoparticle data images, we selected titanium dioxide nanoparticle images captured under scanning electron microscope for particle segmentation experiments. These models achieved better segmentation of the nanoparticle images, with an overall MIoU of 85%. The HRU2-Net† model designed in this paper showed the best performance with an MIoU of 87.37%, exhibiting superior segmentation effects and robust segmentation capabilities. Compared to traditional methods, the deep learning-based semantic segmentation model offers greater efficiency and time savings, and is capable of accurately and meticulously segmenting nanoparticles observed under an electron microscope.

In conclusion, the deep learning-based semantic segmentation model represents a novel tool for the rapid identification and segmentation of a variety of nanoparticles. This development paves the way for new approaches to analyze the morphology of nanoparticles and investigate their properties. Deep learning technology has achieved unprecedented breakthroughs, and the integration of deep learning-based semantic segmentation technology into the field of nanoparticle segmentation has demonstrated significant practical value. Moving forward, we plan to collect a larger dataset of high-quality nanoparticle images, design more advanced semantic segmentation models, and introduce more cutting-edge deep learning techniques.

## Data Availability

The nanoparticle dataset was obtained from the open source project of Bastian Rühle et al., which can be available from the Refs^28^. The datasets used and/or analysed during the current study available from the corresponding author on reasonable request.

## References

[1] Pengyuan Zhu‡, Yifan Kang‡*, Xinglong Li, Haoquan Yu, Tong Liu, Ming Song, Yanan Zhang, Lifan Zhou*, Ping Zhao*b, Wenhuan Huang, UV-modification of Ag nanoparticles on α-MoCx for interface polarization engineering in electromagnetic wave absorption, Nanoscale, 16, 6249–6258 (2024). 10.1039/d3nr05917k 38449440

[2] Maha, M. et al. Nanomaterials: A comprehensive review of applications, toxicity, impact, and fate to environment. *J. Mol. Liquids* **370**, 121046 (2023).

[3] El-Sayed, A. F., Aboulthana, W. M., Sherief, M. A., El-Bassyouni, G. T. & Mousa, S. M. Synthesis, structural, molecular docking, and in vitro biological activities of Cu-doped ZnO nanomaterials. *Sci. Rep.* **14**, 9027 (2024). 38641640 10.1038/s41598-024-59088-2 PMC11031592

[4] Baig, N., Kammakakam, I. & Falath, W. Nanomaterials: a review of synthesis methods, properties, recent progress, and challenges. *Mater. Adv.* **2**, 1821–1871 (2021).

[5] Chu, T., Zhou, L., Zhang, B. & Xuan, F.-Z. Accurate atomic scanning transmission electron microscopy analysis enabled by deep learning. *Res. Article* **17**, 2971–2980 (2024).

[6] Etemad, A., Shafaat, A. & Bahman, A. M. Data-driven performance analysis of a residential building applying artificial neural network (ANN) and multi-objective genetic algorithm (GA). *Build. Environ.* **225**, 109633 (2022).

[7] Sharafeldeen, A., Elsharkawy, M., Alghamdi, N. S., Soliman, A. & El-Baz, A. Precise segmentation of COVID-19 infected lung from CT images based on adaptive first-order appearance model with morphological/anatomical constraints. *Sensors* **21**, 5482 (2021). 34450923 10.3390/s21165482 PMC8399192

[8] Fahmy, D. et al. How AI can help in the diagnostic dilemma of pulmonary nodules. *Cancers* **14**, 1840 (2022). 35406614 10.3390/cancers14071840 PMC8997734

[9] Amin, N. H., Etemad, A. & Abdalisousan, A. Data-driven performance analysis of an active chilled beam air conditioning system: A machine learning approach for energy efficiency and predictive maintenance. *Results Eng.* **23**, 102747 (2024).

[10] Kasgari, A. B. et al. Point-of-interest preference model using an attention mechanism in a convolutional neural network. *Bioengineering* **10**, 495 (2023). 37106681 10.3390/bioengineering10040495 PMC10135568

[11] Aghamohammadi, A. et al. A deep learning model for ergonomics risk assessment and sports and health monitoring in self-occluded images. *Signal Image Video Process.* **18**(2), 1161–1173 (2024).

[12] Ranjbarzadeh, R. et al. A deep learning approach for robust, multi-oriented, and curved text detection. *Cogn. Comput.* **16**(4), 1979–1991 (2024).

[13] Khadraoui, A. & Zemmouri, E. Pyramid scene parsing network for driver distraction classification. *Data Metadata* **2**, 154 (2023).

[14] Sun, Z. et al. A deep learning-based framework for automatic analysis of the nanoparticle morphology in SEM/TEM images. *Nanoscale* **14**, 10761–10772 (2022). 35790114 10.1039/d2nr01029a

[15] Faraz, K., Grenier, T., Ducottet, C. & Epicier, T. Deep learning detection of nanoparticles and multiple object tracking of their dynamic evolution during in situ ETEM studies. *Sci. Rep.* **12**, 2484 (2022). 35169206 10.1038/s41598-022-06308-2 PMC8847623

[16] Sahin, M. E., Ulutas, H., Yuce, E. & Erkoc, M. F. Detection and classification of COVID-19 by using faster R-CNN and mask R-CNN on CT images. *Neural Comput. Appl.* **35**, 13597–13611 (2023). 37213321 10.1007/s00521-023-08450-y PMC10014413

[17] Wang, Z. et al. TESN: Transformers enhanced segmentation network for accurate nanoparticle size measurement of TEM images. *Powder Technol.* **407**, 117673 (2022).

[18] Mill, L. et al. Synthetic image rendering solves annotation problem in deep learning nanoparticle segmentation. *Small Methods* **5**, e2100223 (2021). 34927995 10.1002/smtd.202100223

[19] Kumar Lilhore, U. et al. A precise model for skin cancer diagnosis using hybrid U-Net and improved MobileNet-V3 with hyperparameters optimization. *Sci. Rep.* **14**, 4299 (2024). 38383520 10.1038/s41598-024-54212-8 PMC10881962

[20] Deng, T. et al. Comparison of multi-class and fusion of multiple single-class SegNet model for mapping karst wetland vegetation using UAV images. *Sci. Rep.* **12**, 13270 (2022). 35918459 10.1038/s41598-022-17620-2 PMC9345935

[21] Zhang, X. et al. A ResNet mini architecture for brain age prediction. *Sci. Rep.* **14**, 11185 (2024). 38755275 10.1038/s41598-024-61915-5 PMC11098808

[22] Xuebin Qin, Zichen Zhang, Chenyang Huang, Masood Dehghan, Osmar R. Zaiane, Martin Jagersand, U-Net: going deeper with nested U2-structure for salient object detection, University of Alberta, Canada, arXiv:2005.09007v3 (2022).

[23] Jian Wang*, Xiang Long*, Guowei Chen, Zewu Wu, Zeyu Chen, Errui Ding, U-HRNet: Delving into improving semantic representation of high resolution network for dense prediction, Baidu VIS, arXiv:2210.07140v1, (2022).

[24] Ke Sun, Bin Xiao, Dong Liu, Jingdong Wang, Deep high-resolution representation learning for human pose estimation, Proceedings of the IEEE/CVF Conference on Computer Vision and Pattern Recognition (CVPR), 5693–5703, (2019).

[25] Yuanduo Hong, Huihui Pan, Weichao Sun, Yisong Jia, Deep dual-resolution networks for real-time and accurate semantic segmentation of road scenes, arXiv:2101.06085v2, (2021).

[26] Sebastian Ruder, An overview of gradient descent optimization algorithms, arXiv:1609.04747v2, (2017).

[27] Yi Liu, Lutao Chu, Guowei Chen, Zewu Wu, Zeyu Chen, Baohua Lai, Yuying Hao, PADDLESEG: A high-efficient development toolkit for image segmentation, Baidu Inc., arXiv:2101.06175v1, (2021).

[28] Ruhle, B., Krumrey, J. F. & Hodoroaba, V. D. Workflow towards automated segmentation of agglomerated, non-spherical particles from electron microscopy images using artificial neural networks. *Sci. Rep.* **11**, 4942 (2021). 33654161 10.1038/s41598-021-84287-6 PMC7925552

[29] Connor Shorten & Taghi M. Khoshgoftaar, A survey on image data augmentation for deep learning, Survey paper, Open access, 6, (2019).

[30] Lateef, F. & Ruichek, Y. Survey on semantic segmentation using deep learning techniques. *Neurocomputing* **338**, 321–348 (2019).

[31] Shervin Minaee, Yuri Boykov, Fatih Porikli, Antonio Plaza, Nasser Kehtarnavaz, Demetri Terzopoulos, Image Segmentation Using Deep Learning: A Survey, arXiv:2001.05566v5 (2020). 10.1109/TPAMI.2021.3059968 33596172

[32] Mary L. McHugh, Interrater reliability: the kappa statistic, Biochemia Medica, 22(3), Department of Nursing, National University, Aero Court, San Diego, California (2012). PMC3900052 23092060

[33] A. Khadraoui, E. Zemmouri, Pyramid scene parsing network for driver distraction classification. data and metadata 2, (2023).

[34] S. Jadon, A survey of loss functions for semantic segmentation, arXiv:2006.14822v4, (2020).

[35] Changqian Yu, Changxin Gao, Jingbo Wang, Gang Yu, Chunhua Shen, Nong Sang, BiSeNet V2: Bilateral Network with Guided Aggregation for Real-time Semantic Segmentation, arXiv:2004.02147v1 (2020).

[36] Li, Y., Hu, J., Wen, Y., Evangelidis, G., Salahi, K., Wang, Y., Tulyakov, S., & Ren, J. Rethinking Vision Transformers for MobileNet Size and Speed. Proceedings of the IEEE/CVF International Conference on Computer Vision (ICCV), 16889–16900, (2023).

[37] Jian Wang*, Chenhui Gou*, Qiman Wu*, Haocheng Feng, Junyu Han, Errui Ding, Jingdong Wang†, RTFormer: Efficient Design for Real-Time Semantic Segmentation with Transformer, Baidu VIS & Australian National University (ANU), presented at the 6th Conference on Neural Information Processing Systems (NeurIPS 2022).

[38] Juncai Peng, Yi Liu, Shiyu Tang, Yuying Hao, Lutao Chu, Guowei Chen, Zewu Wu, Zeyu Chen, Zhiliang Yu, Yuning Du, Qingqing Dang, Baohua Lai, Qiwen Liu, Xiaoguang Hu, Dianhai Yu, Yanjun Ma, PP-LiteSeg: A Superior Real-Time Semantic Segmentation Model, Baidu Inc., arXiv:2204.02681v1, (2022).

